# Endoscopic anatomy of the anterior ethmoidal artery: a cadaveric dissection study

**DOI:** 10.1016/S1808-8694(15)30961-7

**Published:** 2015-10-19

**Authors:** Bernardo Cunha Araujo Filho, Raimar Weber, Carlos Diógenes Pinheiro Neto, Marcus Miranda Lessa, Richard Louis Voegels, Ossamu Butugan

**Affiliations:** a Otorhinolaryngologist, residency at HCFMUSP, Member of the ABORL-CCF, PhD student at the Otorhinolaryngology Department HCFMUSP; b Resident Physician; c MD – Federal University of Ceará. Otorhinolaryngology Resident at the University Hospital – University of São Paulo; d PhD in Sciences – Otorhinolaryngology Department - FMUSP, Fellow in Endoscopic Nasal Surgery – University of Graz/Austria, Professor of Otorhinolaryngology – Medical School - UFBA; e Associate Professor - FMUSP; f Associate Professor - FMUSP. Study carried out at the Department of Otorhinolaryngology – University Hospital – Medical School of the University of São Paulo - HCFMUSP

**Keywords:** anatomy, ethmoidal anterior artery, endoscopic, endoscope

## Abstract

**Introduction:**

The anterior ethmoidal artery (AEA) is an important point of anatomical reference in order to locate the frontal sinus and the skull base. Notwithstanding, despite numerous endoscopic studies in cadavers, we still lack an anatomical study on the AEA in the western population.

**Aim:**

to determine reference points used to locate the artery, study its relationship with the skull base and its degree of dehiscence, as well as to study intra and inter individual variations.

**Materials and Methods:**

we dissected the nasal fossae belonging to 25 cadavers.

**Results:**

the average intranasal length of the anterior ethmoidal artery was 5.2 mm. The anterior ethmoidal canal presented some degree of dehiscence in 66.7%. The average distance between the artery middle point to the anterior nasal spine was of 61.72 mm (sd = 4.18 mm); to the lateral nasal wall (nasal axilla) was of 64.04 mm (sd = 4.69mm); and from the anterior axilla to the middle turbinate was of 21.14 mm (sd = 3.25 mm). For all the measures there was no statistically significant measures when both sides were compared (p>0.05).

**Conclusions:**

We concluded that the middle conchae axilla is the most reliable point of reference to locate the AEA.

## INTRODUCTION

The lack of knowledge on endonasal surgical techniques, most specially its anatomy, was the main factor related to the high rate of complications seen associated to this surgical procedure during the 80's^1^. However, today, with greater experience and more knowledge about nasosinusal endoscopic anatomy, acquired through cadaver dissections, the rate of severe complications has reduced considerably^2^. Thus, identifying nasosinusal anatomic structures and knowing its boundaries are essential for both the efficacy and the safety of nasosinusal endoscopic surgeries, regardless of the technique used^3^.

The anterior ethmoid artery (AEA) is an important anatomic point of reference to locate the frontal sinus and the anterior skull base^2^. An unwanted damage to this artery during surgery may cause serious complications, such as intense bleeding, CSF leak, artery retraction towards the intra-orbitary region and, consequently, orbit hematoma and even cerebral infections^4^, ^5^. In rare cases (1%), a surgical treatment is necessary to control posterior epistaxis, by means of artery ligation or cauterization^6^, and in selected cases, with posterior bleeding; the anterior ethmoid artery should also be ligated^7^. In their study about failures in ligating the sphenopalatine artery, Rockey et al.^7^ considered the possibility of always ligating the AEA during the same surgical approach. Woolford et al.^3^ propose an endoscopic procedure for this ligation, and this makes it fundamental to know its endoscopic anatomic location.

Thus, we see that the ethmoid artery correct location is very important in order to recognize structures of difficult access (frontal sinus) and outlining the upper limit during endonasal surgery (skull base). Besides, finding this artery allows us, in some instances, to treat a severe epistaxis site.

Ethnicity and gender have been proven to cause anatomical differences as far as the anterior ethmoid artery is concerned^2^. However, despite many endoscopic studies carried out in cadavers, we still lack an anatomical study of the AEA in the western population, in terms of its relationship with the skull base, in order to normalize its location during endoscopic nasosinusal procedures (ethmoidectomies, frontal sinus surgeries, AEA endoscopic ligation).

The present study aims at studying the endoscopic anatomy of the anterior ethmoid artery, through the dissection of cadaver nasal cavities, identifying reference points for its location and studying its intra and inter-individual variations.

## MATERIALS AND METHODS

This study was approved by the Ethics Committee for Research Project Analysis - CAPPEsq of the Clinical Board of the University Hospital of the University of São Paulo (protocol # 113/04). We dissected 25 cadavers (50 nasal cavities) consecutively at the University Hospital Morgue (SVOC-USP). Cadaver data regarding gender, age, height, weight and race were collected. The cause of death was not taken into account, and we only included individuals above 18 years of age. Notwithstanding, some exclusion criteria were used:

• Past of nasal injury;
• Past of nasosinusal surgery;
• Presence of diseases that altered the anatomy and made it difficult to observe the sphenoid sinus structures (e.g. sinusitis, polyposis etc.).

### Methods

#### Video-documentation system

All procedures were documented by a system comprising a halogenous light source (Konlux-HL2250); a micro-camera (Toshiva IK-CU 43ª); a video screen (SEMP 10”) and a digital video recorder (SONY Mini-DV DCR-TRV 50). This system was taken to the SVOC-USP morgue and used to record all the procedures.

**Figure 1:**
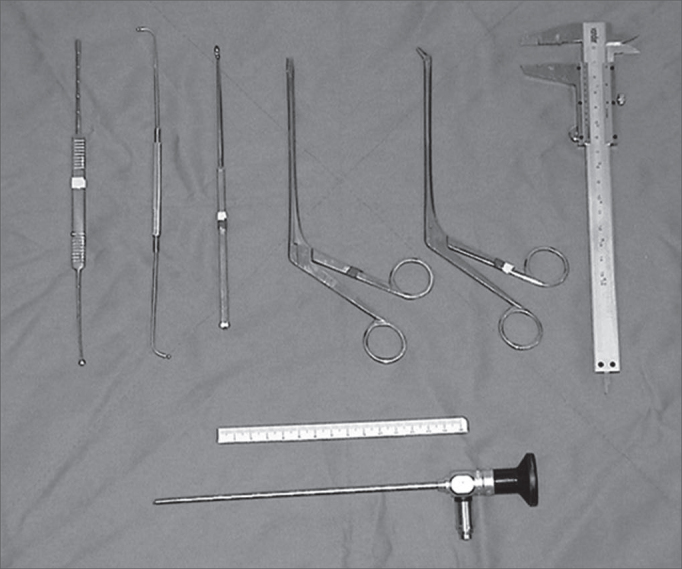
Instruments used for dissection.

Endonasal surgery instruments were used for the dissections (Cottle, angled and straight pressure forceps, cutting forceps, a seeker and a curette for the frontal sinus), a 4mm 0o Storz Hopkinsâ and a 4mm 45o Storz Hopkinsâ endoscopes, a 10mm wide and 100mm long ruler and a pachymeter for the measurements (Figure 1).

#### Dissection technique

**Figure 2:**
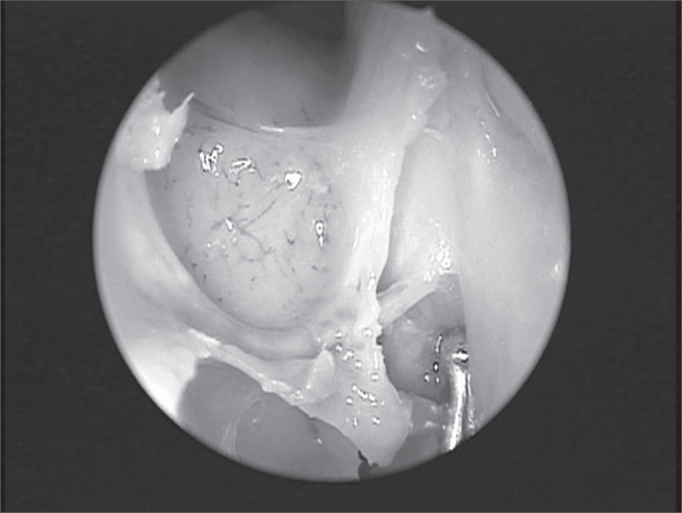
Anterior ethmoid artery in the left nasal cavity during endoscopic dissection in cadaver, depicting the complete dehiscence of the bony canal.

The dissection, always carried out by the same surgeon, comprised the following for each nasal cavity: uncinectomy and anterior ethmoidectomy all the way to the anterior skull base where the anterior ethmoid artery was located. After that, the following were made: removal of the papyraceous wall anteriorly and adjacent to the anterior ethmoid artery; detachment of the papyraceous wall and the periorbital tissue to confirm the AEA identification in the region where it penetrated the papyraceous wall, through the anterior ethmoidal foramen (Figure 2).

**Figure 3:**
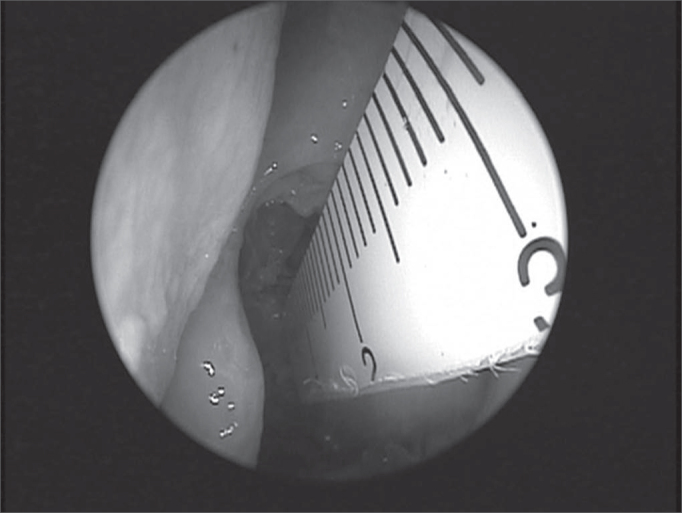
Measure with ruler of the distance between the anterior ethmoid artery and the middle turbinate axilla.

The intranasal artery route was analyzed in each side as to the presence of bone canal dehiscence (total or partial) after removing the nasosinusal mucosa over the artery, using a seeker. Using the ruler, we measured the distances from the middle point of the artery intranasal portion in relation to the skull base, to the middle conchae anterior axilla (anterior border of the middle turbinate insertion on the lateral nasal wall - AXCM), to the upper-medial nostril border (region where the medial and lateral inferior lateral cartilage crura meet - AXN) and to the anterior nasal spine (ENA) (Figure 3). Moreover, its distance to the skull base, classified in 3 groups (< 2.5mm; > 2.5 and < 5mm; > 5mm), and its length along the ethmoid route were checked.

#### Statistical analysis

The data collected were stored in a data base and analyzed using the SPSSâ 10.0 for windows software. The chi-squared, non-parametric test was used for the comparison of the dehiscence prevalence between the sides. In order to analyze measures difference among genders and sides, we used the Mann-Whitney U test. The measures between the sides were correlated using the Pearson linear correlation coefficient. The agreement of dehiscence presence between the sides was analyzed through the Kappa agreement coefficient. “p” values below or equal 0.05 were considered statistically significant. In order to calculate sample and a 95% confidence interval, the confidence grade was of 5% for the non-parametric values.

## RESULTS

Among the cadavers studied, 10 (40%) were males and 15 (60%) were females. Age varied from 39 to 83 years (average: 60.64 ± 12.63 years). Table 1 depicts data regarding ethnicity for the 25 cadavers involved in the study. However, we did not observe statistically significant differences as to the measures observed among the different races studied (p> 0.05).

**Table 1:** Racial distribution.

RAÇA	
Caucasoid	52%
Africanoid	20%
Brown	28%
Total	100%

We studied 50 nasal cavities and in two of them we were unable to locate the anterior ethmoid artery. The anterior ethmoid canal was partially dehiscent in 41.7% of the nasal cavities and fully dehiscent in 25% (Table 2). The canal was intact in 33.3% of the cases. There was no statistical difference, as far as dehiscence was concerned, between the two genders (χ^2^ p= 0.45). The agreement between the right and left sides in relation to dehiscence of the ethmoid canal was week, with a Kappa coefficient of 0.337; the sides were in agreement in only 52% of the cases.

**Table 2:** Dehiscence in the anterior ethmoid canal.

	ETHMOID CANAL		
	Frequence	%	% valid
Dehiscent	12	24,0	25,0
Partially dehiscent	20	40,0	41,7
Intact	16	32,0	33,3
Total	48	96,0	100,0
Not found	2	4,0	

Total	50	100,0	

The distances of the anterior ethmoid artery in relation to the anatomical references in this study are presented on Table 3. The average length of the anterior ethmoid artery intranasal route was of 5.82mm (standard deviation = 1.41mm). The average distance of the artery middle point to the anterior nasal spine was of 61.72mm (sd = 4.18mm); to the AXN was of 64.04mm (sd = 4.69mm); and to the AXCM it was 21.14mm (sd = 3.25mm).

**Table 3:** Anterior ethmoid artery measures in relation to the reference points and according to gender.

Reference point	N	Average Mm	Standard deviation	P (Mann-Whitney U test)
Anterior ethmoid artery length	48	5,82	1,41	0,885
Male	19	5,89	1,62	
Female	29	5,78	1,28	
Distance from the anterior nasal spine	48	61,72	4,18	0,000
Male	19	65,10	1,66	
Female	29	59,51	3,85	
Distance from the anterior nasal axilla	48	64,04	4,69	0,000
Male	19	67,31	2,45	
Female	29	61,89	4,60	
Distance from the middle turbinate anterior axilla	48	21,14	3,25	0,640
Male	19	21,31	2,84	
Female	29	21,03	3,54	

**Chart 1:**
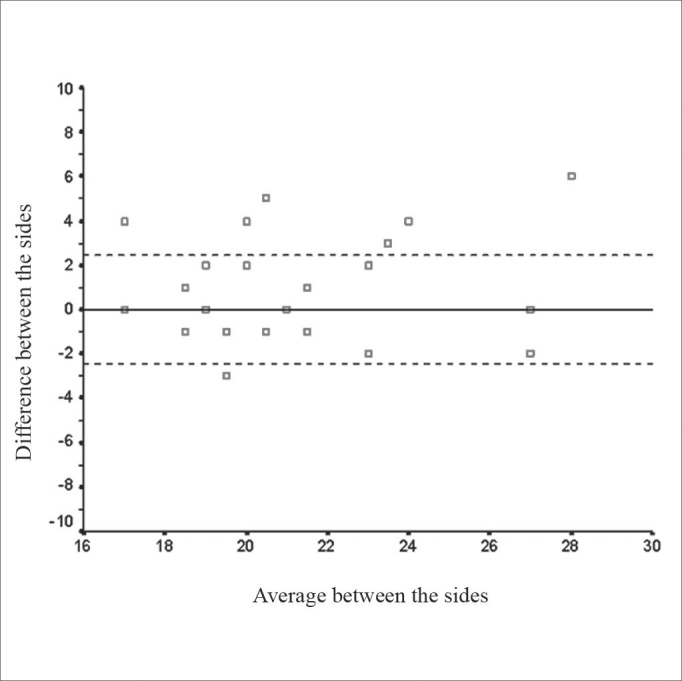
Relationship of the anterior ethmoid artery distance to the anterior insertion of the middle turbinate between the right and left side.

For all the measures, there were no statistically significant differences when the right and left sides were compared (p> 0.05). Such fact was observed in the agreement between the sides as to the distance of the ethmoid artery to the AXCM (Chart 1).

There were statistically significant differences between the genders as to the distance between the artery and the ENA and the AXN (p< 0.0001). Males had longer average distances between the artery and the ENA, and between the artery and the AXN when compared to the measures found in females (Table 3). However, we observed that the distance between the artery and the AXCM did not present statistically significant differences between genders (p= 0.640). Table 3 depicts the averages. The intranasal route length of the anterior ethmoid artery was also not different between genders (p= 0.885).

**Figure 4:**
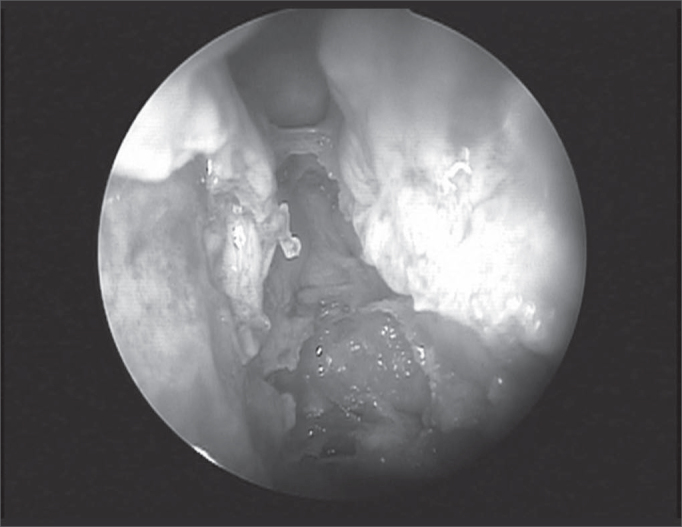
Presence of the anterior ethmoid canal in the nasal cavity crossing the ethmoidal labyrinth at more than 5mm away from the skull base (45 degree endoscope).

**Figure 5:**
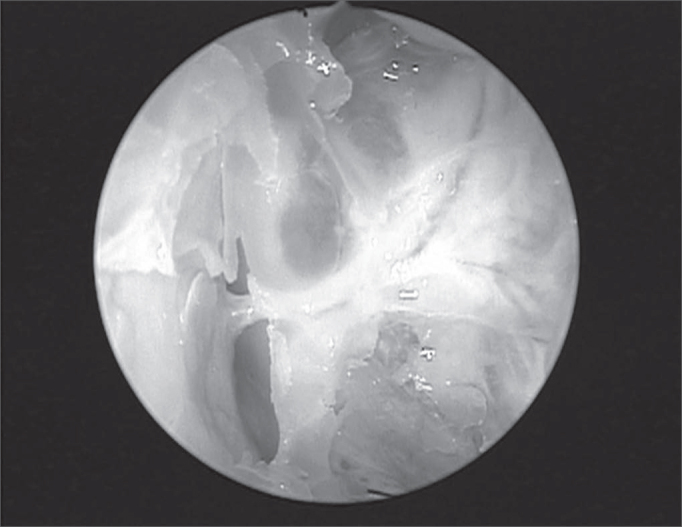
Anterior ethmoid artery crossing the ethmoidal labyrinth at the ceiling of the anterior ethmoid, fully covered by the bony canal.

As to the distance between the artery and the skull base, 83.3% of the arteries were attached to the ethmoid ceiling (Figure 4), 4.1% were between 2.5 and 5mm; and 12.5% were located at a distance greater than 5mm from the skull base (Figure 5). There was no difference between sides (p= 0.383).

## DISCUSSION

In its intranasal route, the anterior ethmoid artery lies inside a bony canal called anterior ethmoidal canal that leaves the orbit through the anterior ethmoidal foramen. This artery is responsible for irrigating anterior ethmoidal cells and the frontal sinus, it branches off into meningeal vessels within the olfactory cleft and it descends to the nasal cavity where it irrigates the anterior third of the nasal septum and the lateral wall of the adjacent nasal cavity4. This artery runs through the ethmoidal ceiling in a diagonal direction, postero-inferiorly (Figure 2) and the site where it penetrates the skull (joint between the cribiform plate and the olfactory cleft lateral lamella) is the region most fragile and prone to injury, causing CSF leaks^8^, ^9^.

A number of studies have concocted techniques to help identify the AEA. Kirchner et al.^8^ studied the AEA anatomy, useful for an external approach, not endoscopic. Stammberger et al.^10^ suggested that the AEA would be located at 1 to 2mm posteriorly to the highest point of the ethmoidal bulla anterior face; while Lund et al.^11^ submit that the frontal recess posterior wall is the reference point for this artery. However, these studies all mention possible ethnical differences, which are not always a rule.

In our study, there was some artery dehiscence in almost two-thirds (66%) of the nasal cavities dissected. Such finding stresses the importance of knowing the exact location of the AEA during endonasal procedures, since dehiscence of the bony canal makes the artery more susceptible to unwanted injuries. Having a canal dehiscence in one nasal cavity does not mean the contralateral artery will also be exposed, since this was true in only 52% of the cadavers. Moon et al.^3^ and Stammberger et al.^8^ presented lower rates of dehiscence in their studies (11.4% and 40%, respectively). The fact that we considered partial dehiscence and racial differences may explain such differences.

We were unable to locate the AEA in two nasal cavities. Lee et al.^2^ considered the absence of the ethmoidal artery; however they did not prove this in their study carried out only among Chinese subjects. Isaacson et al.^12^ studying children skulls, reported anterior ethmoidal foramen absent in 5% of the cases. There are reports of absent AEA and of its route within the skull base bony wall^13^, thus making it impossible to locate, however we do not relate its absence to the latter, since we confirmed its presence in its intra-orbitary route (out of its bony route).

The present study has shown that the anterior ethmoid artery is located, in general, at 21.14mm away from the middle turbinate anterior axilla; at 61.72mm away from the anterior nasal spine; and at 64.04mm away from the upper-medial nostril border. Such measures were very similar to those found by Lee et al.^2^. The same authors described a linear relation between the upper medial nostril border, the middle turbinate anterior axilla and the anterior ethmoid artery (Figure 3), however, we do not agree with such findings. We observed that this straight line met the skull at 3 to 4mm posteriorly to the anterior ethmoid artery (AEA). In their study, Lee et al.^2^ removed the region equivalent to the middle turbinate axilla mucosa, and this could possible bring its measure more anteriorly. In similar studies, Moom et al. and Lee et al. used 0 degree endoscopes in their dissections, and this may impair AEA finding, which we often times were only able to locate using a 45 degrees endoscope. The linear relation is valid; however we must consider the posterior overestimation of this rule.

The measures found in the present study were significantly different between the genders, being greater in males, thus disagreeing from data collected in other dissection studies^2^, ^4^. It was only the distance between the AEA to the AXCM that did not present differences between genders and side, and its standard deviation was the lowest observed among the distances measured in the sample. Thus, we may consider this measure as very useful and reliable in clinical practice to help locate the AEA.

In our study, 12.5% of the AEA were more than 5mm away from the ethmoid ceiling, and such fact was also observed by Moon et al. (14.3%). This allows us to infer that our assessment method was satisfactory for this item, even when we did not use cross sections (sagittal cross sections) (of more precise study) of the head, such as those done by Moon et al. This fact makes ethmoid surgeries more prone to AEA injury. A careful endoscopic exploration, paying attention to the ethmoid ceiling is necessary in order to avoid the possible injuries and irreparable sequels aforementioned.

Ohnishi et al.4 and Lynch considered the possibility of doing a lateral cantotomy to treat possible AEA iatrogenic lesions. However, based on our methodology, we may consider ligating this vessel endoscopically, as advocated by Woolford et al.14, or by skin micro-incision and use of endoscopes, as proposed by Douglas et al.^15^.

## CONCLUSIONS

• The middle turbinate axilla is the most reliable reference to locate the AEA, since it is located in general at 21mm away from the artery, and it did neither show gender nor side differences.
• In 12.5% of the cases in our sample the risk of inadvertently injuring the anterior ethmoid artery during Functional Endoscopic Sinus Surgery was greater because it was located over 5mm off the ethmoid ceiling.
• In 66.7% of the cases, the anterior ethmoid canal presents some degree of dehiscence. Thus, endoscopic sinus surgery in the ethmoid sinus should be careful, establishing the limits in order to avoid severe complications.
